# Is It Feasible to Conduct Post-Tuberculosis Assessments at the End of Tuberculosis Treatment under Routine Programmatic Conditions in China?

**DOI:** 10.3390/tropicalmed6030164

**Published:** 2021-09-10

**Authors:** Yan Lin, Yuqin Liu, Guanghui Zhang, Qinghe Cai, Weihua Hu, Lixin Xiao, Pruthu Thekkur, Jonathan E. Golub, Anthony D. Harries

**Affiliations:** 1 International Union against Tuberculosis and Lung Disease, 2 Rue Jean Lantier, 75001 Paris, France; ylin.consultant@theunion.org (Y.L.); Pruthu.TK@theunion.org (P.T.); 2 International Union against Tuberculosis and Lung Disease, No. 1 Xindonglu, Beijing 100600, China; 3 Infectious Disease Hospital of Heilongjiang Province, No. 1 Jianshe Street, Hulan District, Harbin 150500, China; liuyuqin_ssy@163.com (Y.L.); 15245161620@163.com (W.H.); 4 Number 8 Hospital of Xinjiang Medical University, No. 106 Yanan Road, Urumqi 830000, China; wujie24@unicom.cn; 5 Shantou Institute of Tuberculosis Control and Prevention, No. 1 Honglingjin Road, Shantou 515031, China; gdstjfs@163.com; 6 Xingguo County Institute of Tuberculosis Control and Prevention, No. 26 Yongfu Street, Xingguo 342400, China; WY2437540412@163.com; 7 The Union South-East Asia Office, C-6 Qutub Institutional Area, New Delhi 110016, India; 8 Johns Hopkins Center for Tuberculosis, Johns Hopkins University School of Medicine, Baltimore, MD 21205, USA; jgolub@jhmi.edu; 9 Department of Clinical Research, Faculty of Infectious and Tropical Diseases, London School of Hygiene and Tropical Medicine, Keppel Street, London WC1E 7HT, UK

**Keywords:** tuberculosis, China, post-TB assessment, post-TB symptoms, current cigarette smoking, diabetes mellitus, chest-radiography, 6-min walking test, time taken to complete post-TB assessment

## Abstract

There is growing evidence that a substantial proportion of people who complete anti-tuberculosis treatment experience significant morbidity and mortality which can negatively affect their quality of life. It is suggested that national tuberculosis programs conduct end-of-treatment assessments, but whether this is feasible is currently not known. We therefore assessed whether tuberculosis program staff could assess functional and general health status of patients at the end of treatment in five TB clinics in four provinces in China. There were 115 patients, aged 14–82 years, who completed anti-tuberculosis treatment and a post-TB assessment. There were 54 (47%) patients who continued to have symptoms, the commonest being cough, dyspnea and fatigue. Symptom continuation was significantly more common in the 22 patients with diabetes (*p* = 0.027) and the 12 patients previously treated for TB (*p* = 0.008). There were 12 (10%) current smokers, an abnormal chest X-ray was found in 106 (92%) patients and distance walked in the 6-min walking test (6MWT) ranged from 30–750 m (mean 452 ± 120); 24 (21%) patients walked less than 400 m. Time taken to perform the post-TB assessment, including the 6MWT, ranged from 8–45 min (mean 21 ± 8 min). In 98% of the completed questionnaires, health workers stated that conducting post-TB assessments was feasible and useful. This study shows that post-TB assessments can be conducted under routine programmatic conditions and that there is significant morbidity that needs to be addressed.

## 1. Introduction

Worldwide, tuberculosis (TB) remains a major global public health problem and one of the top 10 causes of death overall [1]. Despite a gradual fall in TB incidence and TB mortality over the last two decades, in 2019 there were 10 million new cases of the disease and 1.4 million deaths [1].

Despite a reported treatment success rate of 85% for people newly enrolled on anti-TB treatment [1], a significant proportion of people who complete anti-TB treatment have post-TB pulmonary complications or extra-pulmonary complications that negatively affect their quality of life [2,3,4].

Available data also suggest that people successfully treated for TB have significantly increased mortality following treatment compared with the general population. In 2004, a study in rural Malawi revealed that 65% of registered TB patients had died within 7 years after anti-TB treatment [5]. In 2019, a group from Canada reported that long-term all-cause mortality in TB patients after they completed treatment was nearly three times higher than in a control group, with most deaths attributable to cardiovascular disease [6]. These findings indicate the need for further research to understand and address the biomedical and social factors that affect the long-term prognosis of this population.

Given the current burden of post-TB disease, morbidity and mortality, patients with TB would merit being assessed for general health status at the end of anti-TB treatment. We have previously argued that an end-of-treatment assessment needs to be carried out within the context of a national TB program (NTP), using a simple check list that documents on going symptoms, co-morbidities, social determinants and lung function, with appropriate actions taken if disability is identified [7,8]. However, most NTPs currently only focus on diagnosis and treatment. How post-TB assessments are best performed and carried out in routine healthcare settings under the NTP and whether healthcare workers find these feasible and useful to conduct are not known.

To address this knowledge gap and draw government attention to post-TB disease and quality of life of TB survivors, we carried out a simple and rapid post-TB assessment at the end of anti-TB treatment under programmatic conditions in TB centers in China. The specific objectives were to determine: (i) whether a simple questionnaire and protocol to evaluate symptoms, signs, laboratory investigations and functional status of TB patients who had successfully completed anti-TB treatment was feasible to be carried out by the established healthcare staff, (ii) the findings of the evaluation in terms of the general health status of the patients at the end of anti-TB treatment and (iii) how long it would take for the healthcare staff to complete the questionnaire and perform a six-minute walking test (6MWT).

## 2. Materials and Methods

### 2.1. Study Design

This was a cross-sectional study carried out within the routine health services in China, and patients were prospectively recruited.

### 2.2. Sites and Setting

**Figure 1:**
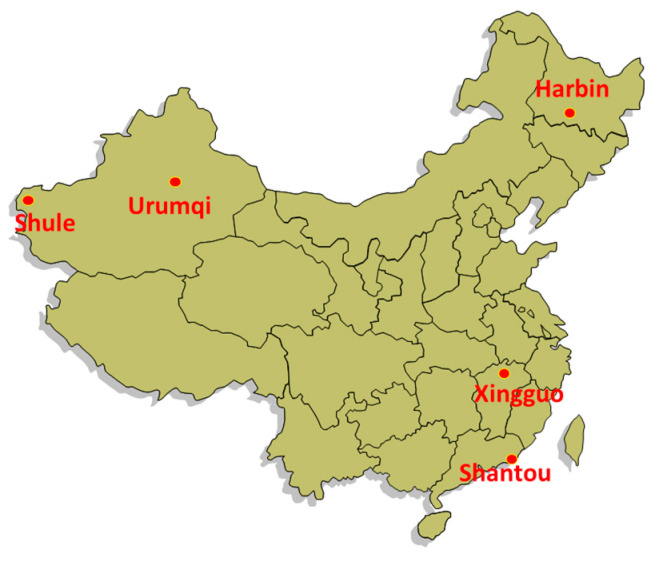
Location of the five health facilities in China.

Five hospitals and outpatient clinics in mainland China were selected. They included the Infectious Disease Hospital of Heilongjiang Province and No. 8 Hospital of Xinjiang Medical University, representing hospitals in the urban areas; Shantou Institute of TB Control and Prevention, representing an urban outpatient clinic; Xingguo County Institute of TB Control and Prevention in Jiangxi Province and the TB Clinic at Shule County Hospital in Xinjiang, representing outpatient clinics in rural areas. The selection of the sites was based on broad geographical coverage, sufficient numbers of TB patients registered each year and willingness of staff to attend to the post-TB work without additional resources. All the sites that were approached agreed to participate. The location of the five sites is shown in Figure 1. Details of the five hospitals and clinics are shown in Table 1. All of the five sites had on-site chest radiograph facilities, a laboratory for blood-tests such as fasting blood glucose, and equipment such as blood pressure machines.

### 2.3. Study Population

Patients included persons aged 14 years and above who had been diagnosed and registered with TB and who had just completed standard anti-TB treatment in one of the five TB hospitals or clinics between April and June 2021 (the second quarter of 2021).

### 2.4. Post-TB on Site Assessment, Data Variables and Evaluation Check List

A simplified evaluation form was developed based on the potential lung damage caused by TB, lifelong comorbidities and measures outlined in the WHO Policy on Prevention of Cardiovascular Disease [9]. The content of the evaluation form is shown in Table 2. The evaluation form was translated to Chinese, printed out in Beijing and sent to the hospitals and clinics for the on-site evaluation. A brief training in how to use the form and how to conduct the 6MWT was conducted in each of the facilities. This form was then used as an evaluation check list by the attending physicians or nurses (who are responsible for TB care) on patients who had either just completed their sixth month of treatment for drug-susceptible TB or their final month for drug-resistant TB. Fasting blood glucose tests were performed after an overnight fast using a standard biochemical analyzer: no point-of-care glucometers were used. Chest radiographs were conducted at the end of treatment and were read by the health facility on-site radiologist.

The 6MWT was performed outdoors. For those hospitals or clinics with a large garden or open square, the attending physician or nurse directed the patients to walk around a track for 6 min using a mobile phone or simple watch to measure the time. For a clinic with no outdoor facility, the 6MWT was performed on the side of the street and patients were directed to walk back and forth on a measured track. Distance walked in meters was measured using a pedometer or a known length of track.

We measured length of time in minutes for the health care staff to complete the questionnaire and perform the 6MWT. However, we did not include length of time to have blood taken for fasting blood glucose or receipt of results by the patient nor length of time for chest radiography or its interpretation by a radiologist. Finally, we invited the healthcare staff to tell us if the post-TB assessments were feasible and useful within their established working environment.

### 2.5. Data Collection, Analysis and Statistics

Individual patient data were received on a monthly basis and cross-checked by the head of each implementing site and the principal investigator (YL). Data were then entered to an Excel file and analyzed. Categorical data were presented as frequencies and proportions and continuous data as means and standard deviations (SD). Comparisons between groups were made using the chi-square test with levels of significance set at 5% (*p* < 0.05).

## 3. Results

Altogether, there were 151 patients who completed anti-TB treatment in the five hospitals/clinics in China between April and June 2021. Of these, 128 (85%) TB patients underwent post-TB assessment, but 13 (10%) were not included in the analysis due to incomplete data. There were, therefore, 115 patients who were assessed and had complete data. Their ages ranged from 14–82 years with a mean (SD) of 44 (±19) years. Details of the number of patients who completed treatment, were assessed and had complete data available in the questionnaire at the five health facilities are shown in Table 3. In 113 (98%) of 115 completed patient questionnaires, healthcare workers stated the assessments were feasible and useful.

The results of the post-TB evaluation are shown in Table 4. There were 54 (47%) patients who continued to be symptomatic after completion of anti-TB treatment, with cough, shortness of breath and fatigue being the most common symptoms. Continuation of symptoms was significantly more common in the 22 patients known to have diabetes (*p* = 0.027) and in the 12 patients who had been previously treated for TB (*p* = 0.008). There were no differences in the proportion with symptoms between those from urban and rural communities (*p* = 0.19) and between those treated in hospitals or in out-patient clinics (*p* = 0.99): patients with drug-resistant TB were mainly treated in hospital, and they had no symptom differences compared with those who had drug-susceptible TB.

An abnormal chest radiograph at the end of treatment was found in 106 (92%) patients. Over one third of patients had either unilateral or bilateral abnormalities, and between 10–20% had shadowing, cavitation or scarring. There were no significant differences in the proportion of patients with chest radiographic abnormalities between those with and without diabetes (*p* = 0.52), between those with drug-susceptible and drug-resistant or multidrug-resistant TB (*p* = 0.41) or between patients presenting to hospitals or outpatient clinics (*p* = 0.91).

The number of meters walked in six-minutes varied from 30–750 (mean 452 ±120). There were 24 (21%) patients who walked less than 400 m during the six minutes. Of these, there were 13 males and 11 females with an age distribution as follows: 14–29 years (6); 30–49 years (3); 50–59 years (4); 60–69 years (4); and 70–82 years (7). There were no significant associations between gender, age group, post-TB assessment symptoms, diabetes status or current smoking status and having a 6MWT less than 400 m (data not shown).

The time taken to complete the post-TB assessment questionnaire, including the 6MWT, ranged from 8–45 min (mean 21 ± 8 min). The mean time to perform the post-TB assessment by staff in Shule County Hospital (mean 26 ± 12 min) was longer than the time in the other sites (mean 15 ± 4 min). These times did not include the performance of the blood-tests and receipt of their results or the chest radiographic examinations and their interpretation.

## 4. Discussion

This is the first report from China to assess the feasibility and findings of conducting post-TB assessments at the end of treatment under routine programmatic conditions. The established healthcare workers in China carried out the additional work within the routine TB services without any extra resources needing to be brought in. In 85% of patients completing treatment, a post-TB assessment was carried out and in the majority of these, questionnaires were completed and the data fully documented, with 20 min on average being needed to complete the task including the 6MWT. Almost all the interviewed healthcare workers felt that these post-TB assessments were useful.

For many decades, most NTPs have traditionally focused on how to increase case detection and how to ensure high treatment success rates, and these aspirations have been fully in line with global strategies to combat and end TB [10,11]. Despite a growing body of evidence from Africa [12,13,14,15], Asia [4], Europe and Latin America [3] indicating a high burden of post-TB lung damage and pulmonary dysfunction, little attention has been paid to this problem. A recent systematic scoping review of 212 international TB guidelines found that only three guidelines mentioned post-TB sequelae and none described how to identify or manage the condition [16].

Our findings, however, indicate that nearly half of the patients successfully completing treatment did not feel healthy and were symptomatic, with cough, shortness of breath and fatigue each occurring in 10–20% of those assessed. These findings are in line with recent studies from Nigeria and South Africa showing that a high proportion of TB patients completing treatment have poor health-related quality of life scores, particularly amongst those aged > 40 years and those with more marked radiographic abnormalities [14,17]. The proportion of our study patients with respiratory symptoms were also in line with findings from Benin and Malawi [12,15]. We did not have the resources to undertake pulmonary spirometry or other more sophisticated tests of lung function to understand the reasons for these continuing symptoms, but it is likely from the published literature that post-TB patients with respiratory symptoms have restrictive pulmonary disease, obstructive pulmonary disease or both [18,19].

In our post-TB evaluation, we investigated for comorbidities and enquired about determinants closely associated with TB. Diabetes mellitus increases the risk of TB by a factor of 2–3 times compared with the general population [20]. Those with diabetes and TB also have a higher risk of death and relapse compared with TB patients who do not have diabetes [21]. Screening of TB patients for diabetes in China has shown that about 12% have concomitant diabetes, with one quarter of those TB patients being newly identified through active screening using fasting blood glucose [22]. Screening of persons with diabetes for TB in China has also shown TB case notification rates to be 3–8 times higher than in the general population [23]. It therefore makes sense to screen for diabetes mellitus at the end of treatment for two reasons. Firstly, this helps to identify persons with new diabetes who may have missed screening opportunities at the start of anti-TB treatment. Secondly, it enables the referral of those with new and those with established diabetes into appropriate diabetes care. We found that 17% of our patients had diabetes diagnosed elsewhere and 3 patients were newly identified with fasting blood glucose ≥ 7.0mml/L requiring referral to diabetes care for confirmatory testing.

Cigarette smoking, excess alcohol consumption and injecting drug use are all associated with an increased risk of TB [24,25,26]. While drinking alcohol and injecting drugs were largely absent in our post-TB patients, 10% were currently smoking. They would benefit from simple attempts to help them quit, and this would not only reduce their risk of recurrent TB but also reduce their risk of non-communicable disease, particularly cardiovascular disease and malignancy.

While there appears to be no association between hypertension and risk of TB [27], we felt it was easy and important to measure the blood pressure at the end of anti-TB treatment. Post-TB patients have a three times higher long-term all-cause mortality compared with the general population, with the leading cause of deaths attributable to cardiovascular disease [6]. Hypertension is an important risk factor for cardiovascular disease and antihypertensive treatment combined with lipid-lowering drugs is effective at reducing long-term mortality [28]. We used the WHO definition of hypertension (systolic blood pressure ≥ 140 mmHg or diastolic blood pressure ≥ 90 mmHg) [29], which equates with stage 2 hypertension as defined in the USA [30]. Few of our patients, however, had hypertension and none needed pharmacologic intervention.

Over 90% of our patients had some degree of chest radiographic abnormality when the chest radiographs were conducted at the end of anti-TB treatment. This proportion seems high, but our findings are in line with those reported from Mexico, Italy and Zimbabwe [3,13]. Similar to those studies, we also found a poor correlation between symptoms and radiographic abnormalities, with many asymptomatic patients having abnormal chest radiographs. Given the resources, time and expense involved in performing a chest radiograph, we need to question whether this is a useful investigation to conduct or not following the completion of anti-TB treatment. One reason for performing chest radiographs is to identify those with residual cavitation who may have chronic pulmonary aspergillosis [31,32]. This condition can be life threatening, for example with aspergilloma and severe hemoptysis. The diagnosis of aspergillosis requires the use of aspergillus-specific immunoglobulin G tests and, if these are positive, the condition can be treated with antifungal drugs.

In the absence of traditional lung function equipment and pulmonary spirometry, we decided to measure the patient’s functional status by a 6MWT. A study in India attested to its simplicity and value in predicting mortality in patients with chronic lung disease [33]. The Indian study included 31 patients with post-TB sequelae: in those patients, the 6MWT showed a median of 192 m walked (range 48–383) in those who subsequently died compared with a median of 335 m walked (range 136–585) in those who survived [33]. In other post-TB studies, the 6MWT has been used to assess functional status, and in Benin about 40% of the patients had an abnormal 6MWT below the lower limit of normal for their age [12]. In high-income countries, there are predicted normal values for the 6MWT which account for the person’s gender, age in years and height in cm [34]. We did not use these reference standards. However, based on a study of 444 healthy subjects aged 40–80 years from seven countries, all but five individuals walked between 400–800 m [35], and we therefore took a walking distance of <400 m as being abnormal. Based on this cut-off, about one fifth of our patients had an abnormal 6MWT, with this abnormality equally divided between males and females and spread over the age range of our study participants.

The strengths of our study were that we attempted to conduct post-TB assessments on consecutive patients completing anti-TB treatment within the routine programmatic setting in China. We used the established staff and no special resource package was introduced to support implementation. Our findings therefore reflect on, and have relevance for, routine TB care in China, demonstrating that it is feasible to conduct such evaluations and that these evaluations do not take excessive time.

There were a number of limitations in the study. The study sample was small and we only selected five clinics. Unfortunately, we did not obtain the reasons for why some patients were not assessed and why 10% of those who were assessed had incomplete questionnaires. Having this information might have given us more insight into feasibility issues. We did not collect information about who had bacteriologically confirmed TB and clinically diagnosed TB, and this might have been useful in assessing post-TB treatment morbidity. In terms of co-morbidities, we did not include the measurement of HIV status and this was because HIV-prevalence in TB patients is <2% in China [1]. We wonder about the accuracy of the lower time limit of 8 min to conduct the post-TB assessment, including the 6MWT, but we had no way at the time of repeating this assessment and therefore took the recording at face value. We only measured the time taken to complete the questionnaire and perform the 6MWT and did not include patient time or health system time in terms of blood-tests and their results or chest radiographs and their interpretation. Finally, our assessment of feasibility and usefulness was based on healthcare workers’ perceptions rather than on any systematic and more objective broad evaluation. 

Despite, these limitations, our study has a number of programmatic implications. Firstly, our post-TB assessment checklist needs to be used elsewhere in the country for more experience in the feasibility of its use and implementation. Attention needs to be paid to having all patients fully assessed on completion of anti-TB treatment, in order for TB patients to potentially benefit from further care. During this expansion, more thought is also needed about its various components. Are we collecting enough information on symptoms, determinants and co-morbidities? Is a chest radiograph needed at the end of anti-TB treatment? Should the 6MWT be better refined in terms of how it is conducted and in terms of generating national reference values for gender, age and height? How in the programmatic context does the 6MWT correlate with more sophisticated lung function tests, and is it a good indicator of the need for pulmonary rehabilitation? In all of these considerations, it is important to balance our wishes for comprehensiveness against the feasibility of busy front-line staff conducting these assessments under routine conditions.

Secondly, while our study did not address or document the further care and assistance that is needed for patients who are still disabled and in need of help at the end of anti-TB treatment, this is an important issue in need of further work. Patients with comorbidities such as diabetes need to be referred back to appropriate care and those with determinants such as smoking need to be helped to quit. Patients with respiratory symptomatology and functional disability should be considered for pulmonary rehabilitation. A Ugandan study showed that a pulmonary rehabilitation package in adults with post-TB lung disease, which was supervised by physiotherapists using aerobic and resistance exercises which were performed later at home, was associated with significant improvements in quality of life, exercise tolerance and respiratory outcomes [36]. Pulmonary rehabilitation programs in post-TB patients in a number of countries in Europe, Africa and Asia have also shown an increase in walking test scores that vary from 35 m to 110 m [37]. These pulmonary rehabilitation packages need to be adjusted to the local context, properly assessed and scaled up for use at the peripheral level. Further clinical research is needed to determine how much additional benefit is gained from airways clearance exercises and use of medications such as inhaled bronchodilators [38]. To ensure that patients complete anti-TB treatment in the best of conditions, we must also seriously consider routinely performing assessments on social determinants, comorbidities and functional status at the time of diagnosis and start of anti-treatment so that issues such as smoking or diabetes mellitus or the need for pulmonary rehabilitation can be addressed earlier.

Finally, this first feasibility study in China paves the way for implementation of the “Fourth 90”, that states “ensuring that 90% of all people successfully completing treatment for TB can have a good health-related quality of life” [39]. The “fourth 90” was proposed in 2019 as an add-on to the three “90-(90)-90” Stop TB partnership targets which were launched in 2015 to help NTPs achieve the WHO End TB goals and targets [11]. The essential activity for the “fourth 90” is a programmatic assessment of TB patients as they complete anti-TB treatment without which post-TB survivors can easily lose touch with the health services. This post-TB assessment is fully in line with the key theme of the Lancet Commission on building a TB-free world [40], where the focus is on patient-centered services to improve the quality and delivery of care not only for TB but also for the multiple comorbidities that ether pre-exist or develop during anti-TB treatment.

## 5. Conclusions

This study, carried out under routine program conditions in patients completing standard anti-TB treatment in five clinics in China between April and June 2021, showed that post-TB assessments were feasible to conduct, they could be conducted in an average time of about 20 min and the health care workers involved found them useful. About half of the patients who were fully assessed had on-going symptoms, the most common being cough, shortness of breath and fatigue. Over 90% had some chest radiographic abnormality at the end of treatment and one fifth had a 6MWT below 400 m. There is significant morbidity amongst TB patients completing anti-TB treatment and this needs to be addressed if we are to honor the Lancet Commission pledge of providing good quality patient-centered care. 

## Figures and Tables

**Table 1:** Details of the five hospitals/clinics that were selected for post-TB assessment of patients who had successfully completed anti-TB treatment.

Variables	Infectious Disease Hospital of Heilongjiang Province	No.8 Hospital of Xinjiang Medical University	Shantou Institute of TB Control and Prevention	Xingguo County Institute of TB Control and Prevention	Shule County TB Clinic in the People’s Hospital
Location	City	City	City	County	County
Population served	31 million	25 million	5.5 million	0.7 million	0.3 million
TB patients in 2020	2003	1600	562	382	197
Main source of patients	Urban/rural	Rural/urban	Urban/rural	Rural	Rural

**Table 2:** End of Treatment TB Assessment Form.

Name of the TB Centre	
**TB Registration Number**	
A. Age	Years
B. Sex	(1) Male (2) Female
C. Living community	(1) Urban (2) Rural
D. Diagnosis	(1) Pulmonary TB (2) Extra pulmonary TB
**SYMPTOMS, COMORBIDITIES, DETERMINANTS**	
E. Do you feel 100% healthy?	(1) Yes (2) No
F. Do you have any symptoms?	(1) No symptoms (2) Cough (3) Shortness of breath (4) Tiredness (Fatigue) (5) Chest pain (6) Other symptoms
G. Do you have Diabetes Mellitus?	(1) Yes (2) No
H. Are you taking anti-DM medication?	(1) Yes (2) No
I. Are you currently smoking?	(1) Yes (2) No
If yes how many cigarettes per day?	Number
J. Do you regularly drink alcohol?	(1) Yes (2) No
If yes, number of bottles (about 640 mL) of beer or the equivalent a day	Number
K. Do you inject drugs?	(1) Yes (2) No
**INVESTIGATIONS**	
L. Systolic Blood pressure	Finding
M. Diastolic Blood Pressure	Finding
N. Fasting blood glucose level	Finding
O. Chest X-ray done	(1) Yes (2) No
P. Chest X-ray abnormal	(1) Yes (2) No
Q. If yes, the abnormality is	(1) Unilateral (2) Bilateral (3) Shadowing (4) Cavitation (5) Scarring (6) Shrinkage (7) Other abnormality
**6-MINUTE WALK TEST**	
Test done	(1) Yes (2) No
R. Meters walked in 6-min	Number
	
S. How long did it take to complete the questionnaire?	Time in minutes
T. Would you be able to fit this sort of questionnaire and assessment into your routine work and do you think it would be useful?	(1) Yes (2) No

**Table 3:** Number of patients who completed anti-tuberculosis treatment and number (%) who were assessed and included in the study in the five healthcare facilities in China from April–June 2021.

Hospitals/Clinics	Number Completing Anti-TB Treatment	Number (%) Assessed at End of Anti-TB Treatment	Number (%) Included in the Study Analysis
Infectious Disease Hospital of Heilongjiang Province	48	45 (94)	42 (93)
No.8 Hospital of Xinjiang Medical University	21	11 (52)	7 (64)
Shantou Institute of TB Control and Prevention	33	30 (91)	29 (97)
Xingguo County Institute of TB Control and Prevention	27	27 (100)	25 (93)
Shule County TB Clinic in the People’s Hospital	22	15 (68)	12 (80)
Total	151	128 (85)	115 (90)

**Table 4:** Results of post-TB assessment in patients completing anti-TB Treatment.

Variables	Number	(%) ^a^
Total patients evaluated	115	
		
Male	70	(61)
Rural	65	(57)
Pulmonary TB	106	(92)
Extrapulmonary TB	9	(8)
Previously treated TB	12	(10)
Multidrug-resistant TB	6	(5)
		
Did not feel healthy and had continuous symptoms	54	(47)
Cough	21	(19)
Shortness of breath	13	(11)
Tiredness and fatigue	20	(17)
Chest pain	9	(8)
Other	8	(7)
		
Known diabetes mellitus diagnosed elsewhere	19	(17)
Newly diagnosed diabetes mellitus during this TB visit	3	(3)
Current smoker	12	(10)
Currently drinking alcohol	1	(1)
Currently injecting drugs	0	(0)
Systolic blood pressure ≥ 140 mmHg	2	(2)
Diastolic blood pressure ≥ 90 mmHg	8	(7)
Fasting blood glucose ≥ 7.0 mmol/L ^b^	15	(13)
		
Any chest radiographic abnormality	106	(92)
Unilateral chest radiographic abnormality	39	(34)
Bilateral chest radiographic abnormality	36	(31)
Chest radiographic shadowing	17	(15)
Chest radiographic cavitation	11	(10)
Chest radiographic scarring	11	(10)
Chest radiographic pulmonary shrinkage	1	(1)
Other chest radiographic abnormality	18	(16)
		
Number of meters walked within 6 min:		
30–299	8	(7)
300–399	16	(14)
400–499	49	(43)
500–599	27	(23)
600–750	15	(13)

^a^ denominator for all percentages is the total number of patients assessed (*n* = 115) ^b^ this includes 12 patients with known diabetes and 3 newly diagnosed with diabetes.

## Data Availability

The data that support the findings of this study are available from the principal investigator (Y.L.—Lin Yan) upon reasonable request.
